# Ultra-Large-Scale Screening of Natural Compounds and Free Energy Calculations Revealed Potential Inhibitors for the Receptor-Binding Domain (RBD) of SARS-CoV-2

**DOI:** 10.3390/molecules27217317

**Published:** 2022-10-28

**Authors:** Lisha Guo, Faryar Zafar, Nawal Moeen, Fahad M. Alshabrmi, Junqi Lin, Syed Shujait Ali, Muhammad Munir, Abbas Khan, Dongqing Wei

**Affiliations:** 1 Zhongjing Chinese Medicine College, Nanyang Institute of Technology, 80 Changjiang Road, Nanyang 473004, China; 2 Nishtar Medical University, Multan 59341, Pakistan; 3 Nawaz Sharif Medical College, Gujrat 50700, Pakistan; 4 Department of Medical Laboratories, College of Applied Medical Sciences, Qassim University, Buraydah 51452, Saudi Arabia; 5 School of Biology and Biological Engineering, South China University of Technology, Guangzhou 510006, China; 6 Center for Biotechnology and Microbiology, University of Swat, Swat 19120, Pakistan; 7 Division of Biomedical and Life Sciences, Lancaster University, Bailrigg, Lancaster LA1 4YW, UK; 8 Department of Bioinformatics and Biological Statistics, School of Life Sciences and Biotechnology, Shanghai Jiao Tong University, Shanghai 200240, China; 9 Zhongjing Research and Industrialization Institute of Chinese Medicine, Zhongguancun Scientific Park, Meixi, Nanyang 473006, China; 10 Peng Cheng Laboratory, Vanke Cloud City Phase I Building 8, Xili Street, Nashan District, Shenzhen 518055, China; 11 State Key Laboratory of Microbial Metabolism, Joint Laboratory of International Laboratory of Metabolic and Developmental Sciences, Shanghai-Islamabad-Belgrade Joint Innovation Center on Antibacterial Resistances, Ministry of Education and School of Life Sciences and Biotechnology, Shanghai Jiao Tong University, Shanghai 200030, China

**Keywords:** SARS-CoV-2, Deltacron, structure-based drugs, molecular simulations, free energy

## Abstract

The emergence of immune-evading variants of SARS-CoV-2 further aggravated the ongoing pandemic. Despite the deployments of various vaccines, the acquired mutations are capable of escaping both natural and vaccine-induced immune responses. Therefore, further investigation is needed to design a decisive pharmacological treatment that could efficiently block the entry of this virus into cells. Hence, the current study used structure-based methods to target the RBD of the recombinant variant (Deltacron) of SARS-CoV-2, which was used as a model variant. From the virtual drug screenings of various databases, a total of four hits were identified as potential lead molecules. Key residues were blocked by these molecules with favorable structural dynamic features. The binding free energies further validated the potentials of these molecules. The TBE for MNP was calculated to be −32.86 ± 0.10 kcal/mol, for SANC00222 the TBE was −23.41 ± 0.15 kcal/mol, for Liriodenine the TBE was −34.29 ± 0.07 kcal/mol, while for Carviolin the TBE was calculated to be −27.67 ± 0.12 kcal/mol. Moreover, each complex demonstrated distinct internal motion and a free energy profile, indicating a different strategy for the interaction with and inhibition of the RBD. In conclusion, the current study demands further in vivo and in vitro validation for the possible usage of these compounds as potential drugs against SARS-CoV-2 and its variants.

## 1. Introduction

The suffering of humanity due to coronavirus disease 2019 (COVID-19), which is characterized by severe respiratory complexities, is linked to an offensive human pathogen known as SARS-CoV-2. This pathogen is a member of the beta (β) coronavirus genus of the subfamily *Orthocoronavirinae* of the family *Coronaviridae* [1]. This disease surfaced in Wuhan, China in 2019, and the pandemic situation was announced by WHO (World Health Organization) due to the rapid range expansion of this pathogen. The situation was further deteriorated by the regular appearance of new variants (e.g., Alpha, Beta, Gamma, Iota, Mu, Delta, Omicron, and finally Omicron-BA.4/5). These new variants evade the immune system and consequently pose a serious threat to humanity [2]. Numerous mutations have been reported in these new variants, and the mutations reported in the spike (S) protein are a matter of concern. These mutations can alter the efficacy of the vaccines, facilitate reinfection, and increase or decrease the severity of the disease [3,4]. The SARS-CoV-2 genome encodes four structural proteins (spike (S), ORF1a/ORF1b, envelope (E), membrane (M), and nucleocapsid (N)) and sixteen non-structural proteins (from NSP1 to NSP16). The proteins such as 3-CLpro, RNA-dependent RNA polymerase (RdRp), and papain-like protease are encoded by the ORF region [5,6] and are considered important druggable targets for the treatment of COVID-19.

To control the current pandemic situation caused by SARS-CoV-2, the global community launched well-coordinated efforts to devise novel strategies, plans, and protocols to search for novel anti-viral drugs and develop an effective vaccine to control COVID-19. So far, the tally of infected persons with this disease has reached 586.96 million, and the estimated death toll has reached 6.43 million worldwide. Previously, influenza and other respiratory diseases were treated with umifenovir or Arbidol, which has a stronger inhibitory mechanism against spike protein. However, it was reported that Arbidol is only effective in mild cases, whereas in severe cases it loses its effectiveness [7,8]. It has been reported that remdesivir can effectively target the RdRP, but it is passing through phase-III clinical trials. Similarly, ritonavir and lopinavir can be used to co-target the RdRp and 3CLpro (the main protease of SARS-CoV-2) to reduce the severity of the disease [9,10,11]. On the other hand, masitinib (a tyrosine-kinase inhibitor) can effectively inhibit 3CLpro and is under clinical evaluation.

The quests for suitable drug targets and safe and effective drugs are crucial to control viral infections, including SARS-CoV-2. Numerous studies have been conducted to control pathogens such as the hepatitis C virus (HCV) and human immunodeficiency virus (HIV) by targeting the glycoproteins that are critical for infection initiation and thus remained well-validated targets for drug designing [12]. Similarly, the cleavage of the pp1a and pp1ab proteases of SARS-CoV-2 is essential for the start of replication and maturation. The inhibition of this step can stop the replication of the virus. Therefore, 3CLpro can be an effective drug target for designing drugs against SARS-CoV-2. The effective drug targeting of 3CLpro, which is highly conserved in all the variants, will be effective and able to produce a prophylactic effect against already-emerged and emerging variants.

Keeping in mind the importance of the SARS-CoV-2 RBD of the spike protein as an effective drug target, we used the virtual screening of an ultra-large drug library and experimental approaches to identify a small molecule. In previous studies, 3CLpro, PLpro, the RBD, RdRp, and other druggable proteins were targeted by using molecular screening approaches to control the viral spread [13,14,15]. The top hits were re-evaluated and validated by using molecular simulation-based approaches. Further validations such as binding free energy calculations, PCAs, and free energy profiles confirmed the pharmacological potentials of these molecules. The current study demands further in vivo and in vitro validation for the possible usage of these compounds as potential drugs against SARS-CoV-2 and its variants.

## 2. Results and Discussion

**Figure 1:**
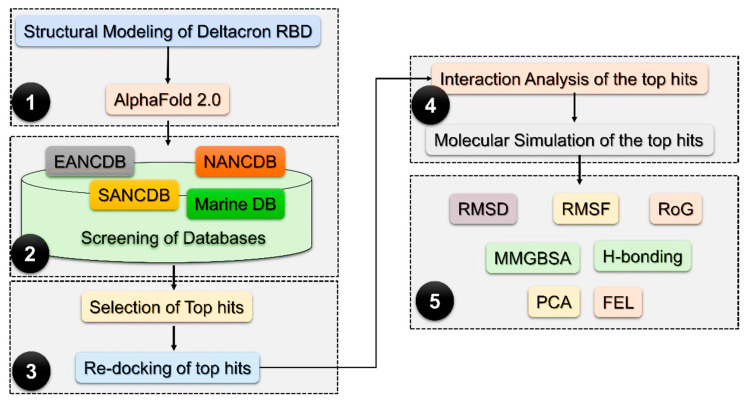
Methodological flow of the whole study. The first step is the modelling of the structure using AlphaFold. In the second step, database retrieval, preparation, and screening are involved. In the third step, the re-evaluation of the top hits from each database is performed, while in the fourth and fifth steps, the validation of the top hits using molecular simulation is performed.

The emergences of ancestral SARS-CoV-2 and now its new variants have generated a distressing situation that has challenged global health, the effectiveness of the marketed therapeutics, and the severity of the disease. For instance, many variants are reported to increase infection rates and frequency. In order to provide a framework for new drug discovery, we used the Deltacron variant as a model virus, which is considered to be the current global health issue. The ability of the Deltacron variant to evade the host immune or vaccine-induced immune response is enabled by its acquired mutations. To date, proteomics- and genomics-based vaccine technologies are employed to design potential vaccine candidates. However, the assimilated unique alterations in the amino acid sequences of the SARS-CoV-2 spike protein provide it with a different ability to evade the immune responses instigated by these vaccines. In this regard, the development of novel drugs or small-molecule inhibitors is of great interest to avoid such problems, i.e., immune evasion. To accelerate the search for potential lead candidates, advanced computational algorithms can fast-track this process. These computational methods have been previously used to design potential inhibitors for SARS-CoV-2. For instance, molecular modelling and simulation-based approaches have been used to target the RBD of the Omicron variant [13]. Similarly, drug repurposing and molecular screening-based approaches also identified potential small molecules for other proteins such as 3CLpro, PLpro, RdRp, S protein, and NRP1 [16,17]. Thus, considering the wide range of applicability of these methods, we also used a large-scale drug screening approach for the RBD of the Deltacron variant. We screened several databases such as the South African natural compounds database (SANCDB), East African natural compounds database, North-East African natural compounds database, and Marine natural compounds database to identify potential small-molecule inhibitors for SARS-CoV-2. The identified potential molecules were validated by using molecular simulation approaches with which the stability, compactness, flexibility, dynamic motions, and binding free energy calculations for each complex were performed. For this purpose, mutations in the RBD of the Deltacron variant were retrieved from the literature. The structure of the RBD was modeled using AlphaFold 2.0. The methodological flow of the study is presented in Figure 1.

**Figure 2:**
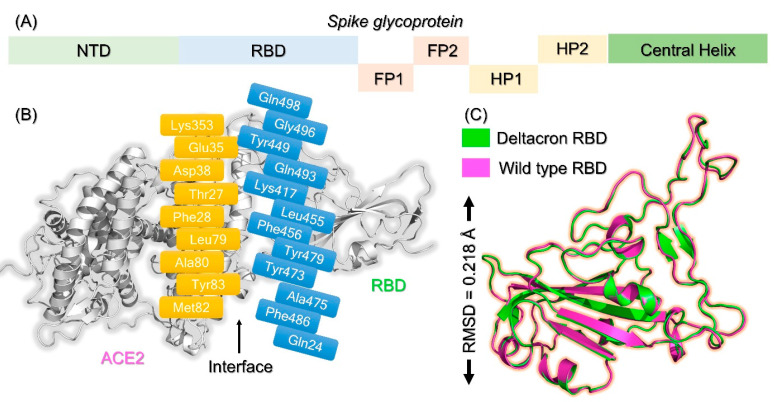
(**A**) Domain organization of the spike protein, (**B**) binding interface, and (**C**) the superimposed structures of the wild-type and Deltacron RBDs.

The structure of the whole S protein with its domain’s organization is shown in Figure 2A, while the RBD structure with its respective mutations is shown in Figure 2B. We also superimposed the structure of the wild-type RBD with that of the Deltacron RBD, which demonstrated an RMS*d* difference of 0.82 Å, thus informing us of the significant structural variations caused by the acquired mutations. The superimposed structures of the wild-type and Deltacron RBDs are shown in Figure 2C. 

### 2.1. Molecular Screening of Natural Compounds Databases

We screened ~55,000 compounds from various databases to identify potential lead molecules for the inhibition of the Deltacron variant of SARS-CoV-2. Among these, 27 compounds reported good docking scores from all the databases, collectively. Among the total twenty-seven compounds, five compounds from the Marine natural compounds database, six compounds from the South African natural compounds database, nine compounds from the North African natural compounds database, one compound from the North-East African natural compounds database, and six compounds from the East African natural compounds database were reported to have competitive docking scores. These top compounds were subjected to further validations by performing another round of screening using AutoDock Vina. The rationale behind the re-evaluation of these compounds was to remove false predictions with smina. The Autodock Vina docking scores ranged from −6.60 to −5.40 kcal/mol. The molecules included *2*-(*2*,*3*-*dihydroxyphenyl*)-*N*-[*2*-(*1H*-*imidazol*-*5*-*yl*)*ethyl*]-*1,3*-*thiazole*-*4*-*carboxamide* (*MNC*) from the CMNPD with docking score −6.60 kcal/mol, (*R*)-*3*’,*5*,*7*-*Trihydroxy*-*4*’-*methoxyspiro* [*2H*-*1*-*benzopyran*-*3*(*4H*),*7*’-*bicyclo* [*4.2.0*]-*octa*[1,3,5]-*trien*]-*4*-*one* (SANC0022) from the SANCDB with docking score −6.32 kcal/mol, and *Liriodenine* from the EANCD and *Carviolin* from the NANCDB with reported docking scores of −5.90 kcal/mol, respectively. These four compounds were observed to be the best among the re-evaluated 27 compounds and were subjected to further investigations such as interactions analysis, dynamic stability, protein’s packing assessment, flexibility indexing, hydrogen bonding, and binding free energy calculations.

**Figure 3:**
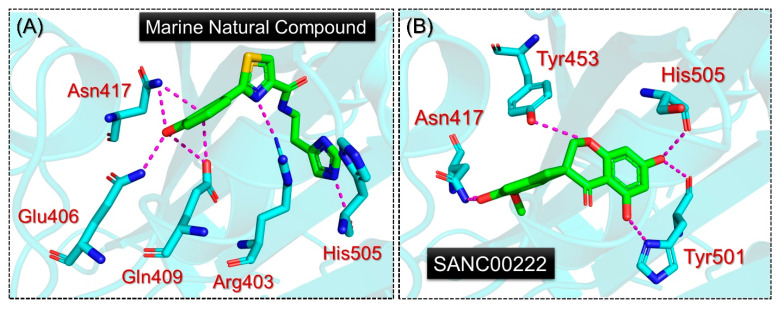
Interaction patterns of MNC and SANC00222 with RBD. (**A**) shows the binding mode of MNC, while (**B**) shows the binding pattern of SANC0022.

The investigation of the binding mode of each of these compounds demonstrated significant results. As shown in Figure 3A, the MNC compound established several hydrogen bonds with the essential mutated residues in the RBD. The residue Arg403 was reported to be involved in the salt-bridge, while Tyr453, Ty495, Ty501, and His505 were reported to have established hydrophobic contacts with the RBD of the Deltacron variant. The MNC compound also established seven hydrogen bonds with the residues Arg403, Gln409, Asn417, Tyr453, and Ser496. Interestingly, most of the targeted residues were the mutated residues of the Deltacron variant, which help to bind with the host receptor more robustly. Moreover, previous studies targeting the RBD of the Omicron variant also reported the same site for inhibition. Using structural modeling approaches, Abbas et. al reported that Tyr453, Arg493, Ser494, Ser496, Tyr501, and His505 are essential residues targeted by the shortlisted compounds and consequently abrogate the RBD-ACE2 complex in the case of the Omicron variant (B.1.1.529) [13]. Moreover, the role of Arg403, Asn417, Tyr501, and His505 in the higher infectivity of other variants has also been revealed, thus showing promising results for the inhibition of the PPI complex [18]. On the other hand, the SANC00222 compound, with two hydrophobic interactions with Tyr501 and His505 and seven hydrogen bonds, also demonstrated a similar interaction pattern to MNC. The hydrogen bonds involved Asn417, Tyr453, Tyr501, and His505 residues. Herein, Tyr453, which is a novel mutation in the Deltacron variant, was observed to be involved in the interaction with the SANC00222 compound. This shows that these two compounds target the key mutated residues by establishing an essential bonding network and preventing the host receptor from interacting with the RBD, which consequently results in no viral entry and infection. The results for SANC00222 are shown in Figure 3B.

**Figure 4:**
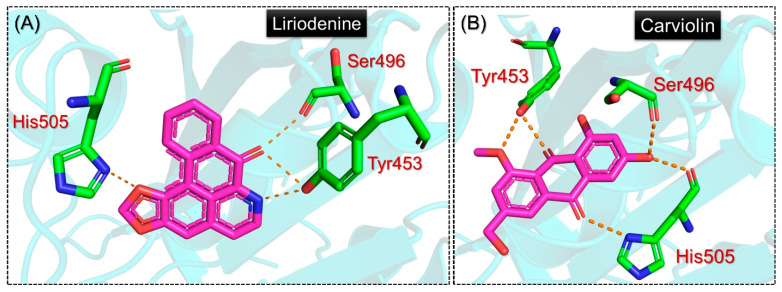
Interaction patterns of Liriodenine from EANDB and Carviolin from NANCDB with RBD. (**A**) shows the binding mode of Liriodenine, while (**B**) shows the binding pattern of Carviolin.

Next, we also evaluated the binding mode of Liriodenine from the EANDB and Carviolin from the NANCDB. The results for these compounds are shown in Figure 4A,B. The Liriodenine compound from the EANDB established a single hydrophobic interaction with Phe497 and four hydrogen bonds involving Tyr453, Ser496, Tyr501, and His505. Interestingly, Tyr453 and Phe497 are among the novel mutations acquired by the Deltacron variant; hence, this shows the potential activity of this compound against the RBD of SARS-CoV-2. The binding mode of Liriodenine from the EANDB is shown in Figure 4A. On the other hand, the Carviolin compound from the NANDB reported a similar docking score to Liriodenine; however, variations in the binding pattern were observed. For the two hydrophobic interactions involving the Tyr495 and Tyr501 residues, five hydrogen bonds were reported, including two with Tyr453 and a single hydrogen bond with Ser496, while His505 also established two hydrogen bonds with this compound. The binding mode of Carviolin with the RBD is shown in Figure 4B. In conclusion, the current findings show promising results by targeting the key essential residues required for direct interaction with the host receptor, thus preventing viral entry into the host cell and consequently reducing the viral infection. The detailed interaction patterns of these compounds along with their docking scores are presented in Table 1.

### 2.2. Structural and Dynamic Stability Assessment of the Top-Scoring Complexes

**Figure 5:**
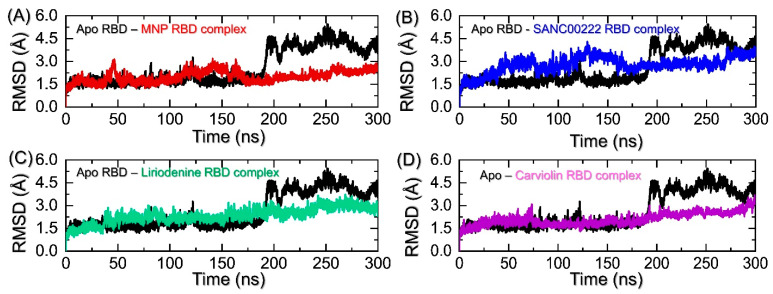
Determination of structural stability in a dynamic environment for the apo-RBD and top complexes. (**A**) shows the RMS*d* patterns for apo-RBD and MNP-RBD complex, (**B**) shows the RMS*d* patterns for apo-RBD and SANC00222-RBD complex, (**C**) shows the RMS*d* graphs for apo-RBD and Liriodenine-RBD complex, while (**D**) represents the RMS*d* graphs for apo-RBD and Carviolin-RBD complex over the course of the 300 ns simulation time.

The computation of RMS*d* in an apo or holo system demonstrates essential information regarding the function of a protein. It is essential in order to determine the binding stability of a small molecule in a complex with a receptor that is usually a protein/druggable target. For this purpose, estimation of the root-mean-square deviation or, shortly, RMS*d* is necessary to comprehend the binding association between the two molecules. The RMS*d* informs us of the stability of a system in a dynamic environment solvated with water molecules and neutralized by other factors in order to essentially understand the effects of the interacting partners. The determination of the dynamic stabilities of small molecules in particular is important in order to gauge their inhibitory potentials and therapeutic efficacies. A small molecule may also destabilize the structural integrity of a receptor to hinder the normal functionality of that particular receptor. Hence, considering the utmost significance of dynamic stability, we herein also used RMS*d* by executing the simulation trajectories to demonstrate the stability of each complex over the course of the simulation time. The calculated RMS*d* for each complex is shown in Figure 5A–D. The apo-RBD shown in Figure 5A reveals a similar RMS*d* pattern as the RMS*d* pattern of the wild type during 1–180 ns, and then abruptly increased. Each complex demonstrated comparatively more stable dynamic behaviors throughout the simulation compared with the apo system. The snapshots at 190 ns, 230 ns, 250 ns, and 300 ns from the apo system were retrieved and superimposed onto the native conformation. The comparative structural investigation revealed that three regions, i.e., 333–350, 470–494, and 510–526, demonstrated significant structural perturbation, which is a major deviation in the RMS*d*. More interestingly, the deviation that occurs between 470 and 494 signifies the important region required for direct interaction with the host receptor. The two important loops reside in this region that is essential for binding. Furthermore, the transition of essential secondary structure elements was also observed. The superimposed structures at these time intervals are shown in Supplementary Figure S1. The RMS*d* of the MNC compound initially increases gradually and attains equilibrium at 50 ns. During the 300 ns simulation, no significant deviation was recorded; however, a minor structural perturbation can be seen between 75 and 85 ns, and then a flattened graph continues until 300 ns. The minor perturbation could be due to the loops distributed in the RBD, which was also witnessed in other studies targeting the hACE2-RBD interface [13]. The RMS*d* is seen to show variations until 175 ns. However, after this, till the end of the simulation (300 ns), a flat graph can be seen, which shows the stabilized binding of the MNC compound. Moreover, the SANC00222 complex with the RBD reports a comparatively higher RMS*d* but is stable. The graph shows an initial increase in the RMS*d* during the first 50 ns, and then an increase/decrease pattern until 135 ns is observed. Although a fluctuation between 51 and135 ns can be seen, it does not demonstrate instability, as it is very minor and the lower RMS*d* is regained afterward. The RMS*d* after 135 ns decreases continuously and follows the same trend until 200 ns. This trend continues until 300 ns with no significant structural perturbation, thus demonstrating a comparatively unstable dynamic compared with MNC. This, consequently, shows that although an increase in the RMS*d* is observed, overall, the complex reports stable dynamics and thus produces an inhibitory potential to abstain from the hACE2-RBD interaction. Interestingly, the two other complexes, i.e., Liriodenine- and Carviolin-RBD, demonstrated more stable dynamics than the first two complexes. The Liriodenine complex reaches equilibrium at 25 ns, followed by a smaller deviation at 45–50 ns, then continues to follow a uniform pattern until 115 ns. The RMS*d* then increases a little, although with no structural perturbation, and then decreases back down after reaching 165 ns. A stable and straight graph is then observed until 200 ns. Moreover, the Carviolin complex reaches stability at 1.5 Å and attains equilibrium at 15 ns. Except for a minor deviation between 70 and 75 ns, this complex demonstrates stable behavior until 200 ns. This complex was observed to be the most stable among the four complexes. For all the complexes, the structures reach stability at 1.5–2.5 Å, and the average RMS*d* was calculated to be 1.5–2.5 Å. The Liriodenine and Carviolin complexes during the last 100 ns (201–300 ns) further continue the stability pattern and report stable dynamics. The RMS*d*s of all the complexes are shown to converge with the wild type, which shows that all the complexes attain a similar conformational configuration, which consequently shows the effect of the ligand binding in the later part of the simulation. This demonstrates that the ligand-bound complexes had already reached stability and the 300 ns simulation was enough to reveal the pharmacological properties of these compounds. Overall, these findings show the stable binding and dynamic behaviors of each molecule in a complex with RBD, thus favoring the pharmacologically essential features for the inhibition of SARS-CoV-2.

### 2.3. Assessing the Structural Packing in a Dynamic Environment

**Figure 6:**
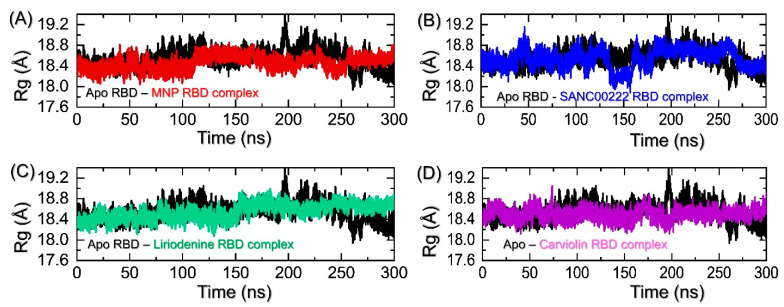
Determination of structural packing in a dynamic environment for the apo-RBD and top complexes. (**A**) shows the R*g* patterns for apo-RBD and MNP-RBD complex (**B**) shows the R*g* patterns for apo-RBD and SANC00222RBD complex, (**C**) shows the R*g* graphs for apo-RBD and Liriodenine-RBD complex, while (**D**) represents the R*g* graphs for apo-RBD and Carviolin-RBD complex over the course of the 300 ns simulation time.

Next, we evaluated the structural compactness of each protein in a dynamic solution as the radius of gyration (R*g*). R*g* may provide essential information regarding the kinetics, inhibition, and thermodynamics of a protein that may generate new insights into the biological processes. It may, alternatively, provide information regarding the binding and unbinding events that occurred during the simulation. Interrelationships between some structural, thermodynamic, and kinetic parameters of proteins have been reported in recent years [19]. Keeping the high importance of R*g* in the biological context herein, we also calculated R*g* as a function of time using 15,000 structural frames from the 300 ns simulation. The R*g* pattern for the wild type demonstrates a uniform graph that converges with other complexes, and the average R*g* was reported to be 18.60 Å. As presented in Figure 6A, the R*g* graph follows the same pattern as the RMS*d* graph where a gradual increase over the time was observed. The initial R*g* increases gradually between 0 and 35 ns, and then until 100 ns, smaller deviations are observed. Afterward, the R*g* slightly increases with time, thus implying that binding and unbinding events occurred during the simulation. For the MNP-RBD complex, the average R*g* was calculated to be 18.50 Å. On the other hand, the SANC00222 R*g* results also strongly corroborate with the RMS*d* results. The R*g* during the first 50 ns demonstrates a uniform pattern fluctuating between 18.40 and 18.60 Å and then increases abruptly, reaching 19.0 Å. The R*g* decreases back down and continues to follow the same pattern with minor deviations between 51 and 135 ns. Further decline in the R*g* value is observed particularly between 140 and 160 ns and then, again, reaches the average level of 18.50 Å. The potential rationale behind the increase and decrease in the protein size is that it is due to the free movement of the three loops that are present at the interface of the RBD required for direct interaction with the host receptor ACE2. The R*g* graph for the SANC00222-RBD complex is shown in Figure 6B. Interestingly, the R*g* graphs for Liriodenine and Carviolin also present the same pattern as those of the RMS*d*s. These two complexes demonstrate stable R*g* patterns and lower average R*g* values. The Liriodenine-RBD complex starts at 18.40 Å, following the trend till 40 ns, and then minor deviations between 41 and 60 ns are recorded. Subsequently, the R*g* is recorded as decreasing until 110 ns, and then a gradual increase is observed. Though the R*g* values slightly increase, no significant deviation is experienced until the end of the simulation. On the other hand, the Carviolin-RBD complex reports higher R*g* values between 40 and 80 ns and between 170 and 185 ns. The graph is observed to be straight. For these two complexes, the average R*g* values were calculated to be 18.60 Å and 18.73 Å, respectively. The R*g* graphs for Liriodenine and Carviolin are shown in Figure 6C,D. In the extended simulation, no significant deviation was observed till the end of the simulation, which shows that these complexes had already reached stability, and the simulation time was enough to conclude the findings. Overall, these results show that either due to the larger movement of the interface loops or the binding/unbinding of these small molecules, they experienced different interaction paradigms during the simulation.

### 2.4. Indexing the Residual Flexibility

**Figure 7:**
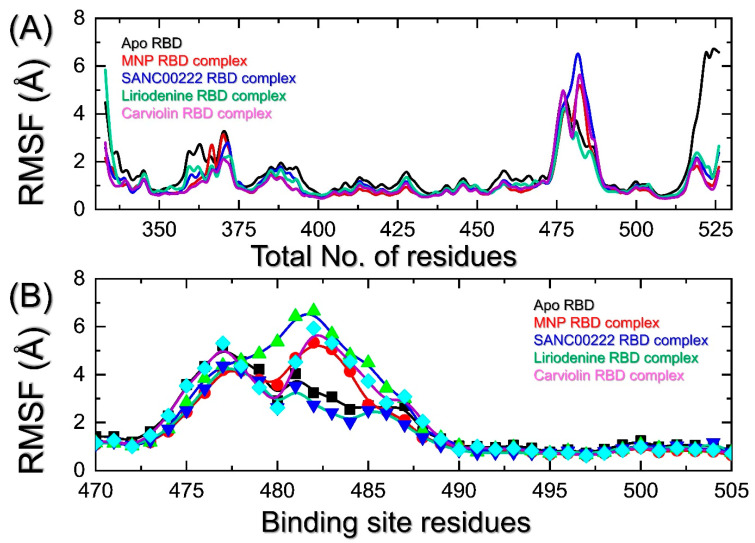
Calculation of residues’ flexibilities in a dynamic environment. (**A**) shows the RMS*f* patterns for all the complexes, while (**B**) show the RMS*f* pattern for region 470–505.

The residual flexibility has an important association with the kinetics and functionality of biological molecules. This approach can be used to determine essential residues that play a significant role in molecular recognition, bio-catalytic mechanisms, manual protein designs, and in the coupling of two or more proteins/interacting partners. In this regard, conformational variations that span a wide variety of amplitude scales are typically linked to protein function. Protein dynamics have been shown to be crucial; thus, knowing about protein flexibility is just as important as knowing about protein structure when it comes to understanding a protein’s function and to improving drug development [20]. Herein, we indexed the flexibility of each residue during the course of the simulation. As presented in Figure 7A, all the complexes show a similar pattern of flexibility for most of the regions. A minimal fluctuation for most of the residues is observed. However, regions such as 358–375 and 473–500 report higher fluctuations. The region 510–525 also reports a higher fluctuation. The residues 470–500 comprise the three important loops required for direct interactions with the host receptor ACE2 and demonstrate higher fluctuations. To determine the flexibility of these loops, the residue flexibility was calculated and is shown in Figure 7B. It can be seen that all the regions, particularly 473–490, exhibit higher fluctuations which, consequently, differentiates the dynamic behavior of each complex induced by the binding of each small molecule.

### 2.5. Analysis of the Hydrogen Bonding during Simulation

**Figure 8:**
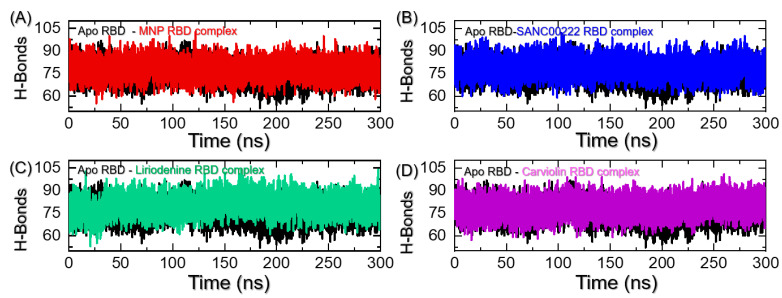
Hydrogen-bonding calculation for each complex during the simulation time. (**A**) shows the H-bonds patterns for apo-RBD and MNP-RBD complex, (**B**) shows the H-bonds patterns for apo-RBD and SANC00222-RBD complex, (**C**) shows the H-bonds graphs for apo-RBD and Liriodenine-RBD complex, while (**D**) represents the H-bonds graphs for apo-RBD and Carviolin-RBD complex over the course of the 300 ns simulation time.

The inhibitory properties of the interacting compounds can be comprehended better by quantifying the hydrogen bonds between the protein and drug molecules. One of the critical elements that define the binding drug’s inhibitory actions is hydrogen bonding. The binding affinity of the interacting molecules changes as the number of hydrogen bonds fluctuates. The estimation of the hydrogen-bonding paradigm has previously been utilized in order to demonstrate how the emerging SARS-CoV-2 variants preferentially impact the binding with the hACE2 receptor by changing the hydrogen-bonding network Moreover, it has also been used in the discovery of novel drugs that target the receptor-binding domain (RBD) of the Omicron variant [13]. Firstly, we calculated the average number of hydrogen bonds in each trajectory to see how the bonding was affected throughout the simulation time. As presented in Figure 8A–D, the hydrogen bonding demonstrates changes over the course of the simulation time in each complex. In each complex, the average numbers of hydrogen bonds were calculated to be 75 in apo-RBD, 80 in MNP-RBD, 78 in SANC00222-RBD, 802 in Liriodenine-RBD, and 79 in Carviolin-RBD. This shows that the additional bonds were probably due to the binding of ligands with the RBD interface.

**Figure 9:**
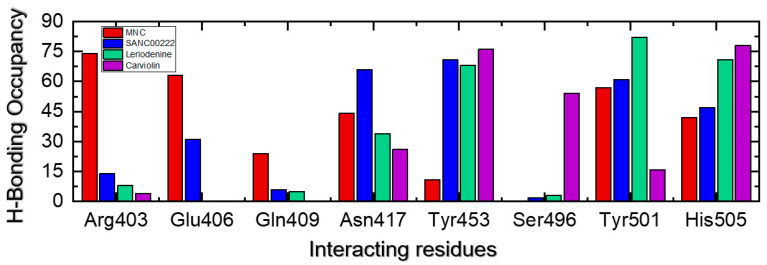
Hydrogen-bonding occupancy calculation for each complex during the 300 ns of the simulation time period.

We also determined the percentage half-life of each interaction during the simulation, as presented in Figure 9. The essential contacts established with Arg403, Glu406, Gln409, Asn417, Tyr453, Ser496, Tyr501, and His505 were monitored in each trajectory. For these residues, the percentage occupancies were calculated to be 74% (Arg403), 63% (Glu406), 24% (Gln409), 44% (Asn417), 11% (Tyr453), 0% (Ser496), 57% (Tyr501), and 42% (His505) for MNC. The calculations of the hydrogen-bonding occupancies for the SANC0022-RBD complex were revealed to be 14% (Arg403), 31% (Glu406), 6% (Gln409), 66% (Asn417), 71% (Tyr453), 2% (Ser496), 61% (Tyr501), and 47% (His505). On the other hand, for these residues, the percentage occupancies were calculated to be 8% (Arg403), 0% (Glu406), 5% (Gln409), 34% (Asn417), 68% (Tyr453), 3% (Ser496), 82% (Tyr501), and 71% (His505) for the Liriodenine-RBD complex. Moreover, the Carviolin-RBD complex demonstrated hydrogen-bonding percentages of 4% (Arg403), 0% (Glu406), 0% (Gln409), 26% (Asn417), 76% (Tyr453), 54% (Ser496), 16% (Tyr501), and 78% (His505) during the simulation. This shows that the interactions with the key residues remained sustained, which consequently produces pharmacological potentials for targeting the RBD of the Deltacron variant.

### 2.6. Gibbs Free Energy Calculation to Re-Evaluate the Best Conformations

The pharmacological effects of any particular drug/small molecule can be pre-clinically validated by predicting the accurate binding conformation and re-evaluating the binding free energy using the MM/GBSA or MM/PBSA methods. These methods are the most popular and reliable methods for re-ranking a potent inhibitor against a particular receptor. They are virtually reasonable and have the advantage over other rational methods. Hence, in view of its accuracy and applicability, we also considered using MM/GSBA to calculate the binding free energy for each complex. In the case of the MNP-RBD complex, the vdW energy was computed to be **−41.11 ± 0.11** kcal/mol, the electrostatic energy was computed to be **−10.34 ± 0.10** kcal/mol, while the total binding energy was estimated to be **−32.86 ± 0.10** kcal/mol. On the other hand, for the SANC00222-RBD complex, the vdW was calculated to be **−29.96 ± 0.10** kcal/mol, the electrostatic energy was estimated to be **−13.31 ± 0.13** kcal/mol, while the total binding free energy was computed to be **−23.41 ± 0.15** kcal/mol. Moreover, the Liriodenine-RBD complex demonstrated a vdW of **−31.73 ± 0.08** kcal/mol, an electrostatic energy of **−8.12 ± 0.08** kcal/mol, while the total binding free energy was estimated to be **−34.29 ± 0.07** kcal/mol. Finally, the total binding free energy was reported to be **−27.67 ± 0.12** kcal/mol for the Carviolin-RBD complex, while the vdW and electrostatic energies were calculated to be **−28.98 ± 0.13** and **−6.54 ± 0.09** kcal/mol, respectively. This shows that the four hits could inhibit the RBD more strongly and require further in vivo and in vitro validation for their possible usage as potential drugs against SARS-CoV-2 and its variants. The binding free energy results are shown in Table 2.

### 2.7. Clustering of Protein Motion in Trajectories

**Figure 10:**
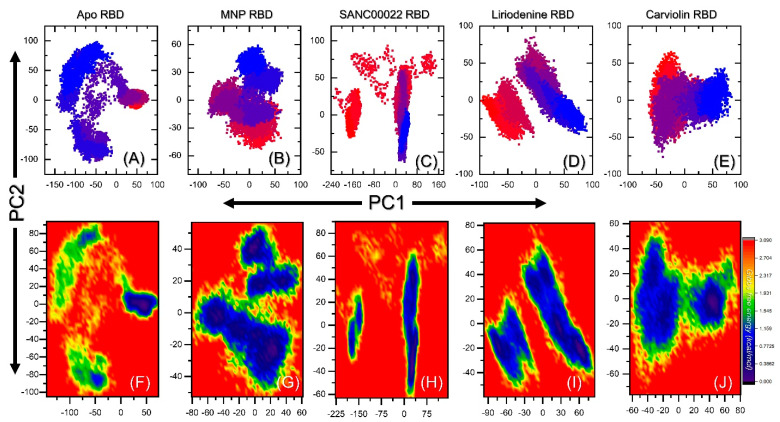
(**A**–**E**) Principal component analysis and (**F**–**J**) free energy landscape profiles of the hits.

For each complex, a PCA analysis was conducted to group similar trajectories and conformations. This method has been widely applied to filter large concerted motions from the set of structures produced by experiments and molecular computations. As shown in Figure 10A–E, each drug reports varied dynamic motions of 47%, 45%, 48%, 39%, and 44% motion of the total internal motion by the first three eigenvectors of each complex. In the illustration, the two colors orange and blue stand in for the two conformations, while purple stands in for the two transition phases. In conclusion, this demonstrates that the final hits exhibit distinct internal motions, indicating their different strategies for the interaction with and inhibition of the RBD.

### 2.8. Free Energy Landscape Analysis

Next, the conformational alterations induced by the binding of these small molecules were further explored by utilizing the free energy landscape. The constructed FEL maps for all the complexes are presented in Figure 10F–J. For each complex, the conformational states are separated by a subspace. For each complex, the free energy profiles are altered, and the overall results reflect the conformational and dynamic changes that favor the bindings of the identified hits to the respective sites.

## 3. Conclusions

In conclusion, the current study used structure-based methods to target the RBD of the recombinant variant (Deltacron) of SARS-CoV-2. From the virtual drug screenings of various databases, four hits were identified as potential lead molecules. Key residues were blocked by these molecules with favorable structural dynamic features. The binding free energies further validated the potentials of these molecules. Further validations such as binding free energy calculations, PCAs, and free energy profiles confirmed the pharmacological potentials of these top hits. The current study demands further in vivo and in vitro validation for the possible usage of these compounds as potential drugs against SARS-CoV-2 and its variants.

## 4. Materials and Methods

### 4.1. Structures Retrieval and Molecular Screening

The accession ID 6M0J was used to obtain the 3D structure of the SARS-CoV-2 spike glycoprotein from the Protein Databank [21]. AlphaFold 2.0 in Google colab was used to model the structure of the RBD of the Deltacron variant. The South African natural compounds database (SANCDB), East African natural compounds database, North-East African natural compounds database, Chinese medicine database, and Marine natural compounds database were searched for the retrieval of small molecules, and then the Open Babel and FAF-drugs4 web tools were used for the preparation and estimation of the Lipinski’s rule violation [22,23]. The compounds which did not follow the R5 rules were removed, and the remaining compounds were processed for molecular screening. The Smina docking tool with the AutoDock scoring function was utilized for the initial virtual molecular search. It has certain advantages, such as its supporting multi-ligand files, various ligand molecular formats, and additional energy terms, such as desolvation and electrostatics energies [24,25]. Moreover, the automatic box creation option makes it more feasible for users. The active site was identified on the basis of the previous literature, and the residues were crosschecked for accuracy. The 50 top hits were re-docked for the removal of any false-positive predictions. 

### 4.2. Molecular Dynamics Simulation of Protein-Ligand Complexes 

The inhibitory features of potential lead molecules and the validation of dynamic behaviors were assessed with the help of molecular simulation. The complexes of compounds and the RBD (Deltacron variant) were subjected to the AMBER21 package for estimation of tier flexibility, stability, and packing. The coordinates, topology, and solvation of the drugs were achieved with an antechamber algorithm and optimal point charge (OPC) water model, respectively. The addition of Na+ counter ions was essential for neutralization purposes. For improved and optimized structures, the minimization of each complex was performed for a cumulative 20,000 steps. After improvement, the complexes were subjected to heating and equilibration for 50 ns. The particle mesh Ewald (PME) algorithm was employed for long-range contacts, whereas van der Waals (vdW) interactions for contacts within a range of 1.4 nm and Columbic interactions for short-range contacts were considered. A total run of 1500 ns and a complex-production run of 300 ns were performed with a time step of 2 fs. CPPTRAJ and PTRAJ were used to obtain the trajectories of each simulation [26]. A root-mean-square deviation (RMSD) analysis for the function of time was performed to check the structural stability by using the following equation:

(1) $$RMSD=\sqrt{\frac{\sum d^{2}i=1}{N_{atoms}}}$$

where *d* denotes the position difference between atoms, and *i* is used for the original and superimposed structure. On the basis of the *B-factor*, root-mean-square fluctuation (RMSF) was calculated and it is the most essential constraint for the calculation of flexibility for all protein residues. RMSF values can be obtained with the following equation:

(2) Thermal factor or B-factor=[(8π×2)/3] (msf)

### 4.3. The Binding Free Energy Calculations

The complexes’ binding free energies were estimated using the MMPBSA.PY script [27]. It calculates each and every energy term that contributes to the total binding energy. We used the whole simulation trajectories for the binding free energy calculations. For all the complexes, free energy was calculated with the following equation:

(3) $$\Delta G_{bind}=\Delta G_{complex}-\left[\Delta G_{receptor}+\Delta G_{ligand}\right]$$

Free binding energy is represented by “Δ*G_bind_”* in the equation. The specific energy terms that contributed to the total free energy were obtained with the following equation:

(4) $$G=G_{bond}+G_{electrostatic}+G_{vdW}+G_{polar}+G_{non\text{-}polar}$$

### 4.4. Principal Component Analysis (PCA)

A PCA was performed to capture the motion trajectories with the help of the CPPTRAJ package. With this method, high-amplitude motions were mapped by considering the eigenvalues of the principal motions [28]. From the obtained trajectories, the PC1 and PC2 high-amplitude fluctuations were selected for plotting against each other.

### 4.5. Free Energy Landscape (FEL)

The two PCs (PC1 and PC2) were used for the contour map construction in order to obtain the lowest energy state (metastable state) and also show the lowest conformations obtained by each complex. The subspaces between the states represent the transition states [29]. The Gromacs module *g_sham* was used for the free energy landscape (FEL). *PC*1 and *PC*2 were used for the FEL calculations by using the following equation:

(5) $$\Delta G\left(PC1,\;PC2\right)=K_{B\;}\ln P\left(PC1,\;PC2\right)$$

## Figures and Tables

**Table 1:** Top hits from different databases along with the interaction types, including hydrophobic, hydrogen bonding, and docking scores.

Complexes	Salt/Hydrophobic Interactions	Hydrogen Bonding	Docking Scores (kcal/mol)
***2*** **-(** ***2*** **,** ***3*** **-** ***dihydroxyphenyl*** **)-** ***N*** **-[** ***2*** **-(** ***1H*** **-** ***imidazol*** **-** ***5*** **-** ***yl*** **)** ***ethyl*** **]-** ***1*** **,** ***3*** **-** ***thiazole*** **-** ***4*** **-** ***carboxamide***	Arg403, Tyr453, Ty495, Ty501, His505	Arg403, Gln409x2, Asn417x2, Tyr453, Ser496	**−6.6 kcal/mol**
***SANC00222***	Tyr501, His505	Asn417x2, Tyr453, Tyr501x2, His505x2	**−6.3 kcal/mol**
***Liriodenine***	Phe497	Tyr453, Ser496, Tyr501, His505	**−5.9 kcal/mol**
***Carviolin***	Tyr495, Tyr501	Tyr453x2, Ser496, His505x2	**−5.9 kcal/mol**

**Table 2:** Binding free energy calculations using MM/GBSA approach.

Parameters	MNP-RBD Complex	SANC00222-RBD Complex	Liriodenine-RBD Complex	Carviolin-RBD Complex
**VDWAALS**	−41.11 ± 0.11	−29.96 ± 0.10	−31.73 ± 0.08	−28.98 ± 0.13
**EEL**	−10.34 ± 0.10	−13.31 ± 0.13	−8.12 ± 0.08	−6.54 ± 0.09
**EGB**	23.29 ± 0.10	22.96 ± 0.13	7.74 ± 0.10	10.45 ± 0.08
**ESURF**	−4.69 ± 0.14	−3.10 ±0.08	−2.18 ± 0.90	−2.61 ± 0.01
**DELTA G gas**	−51.46 ± 0.17	−43.27 ± 0.18	−25.86 ± 0.13	−28.52 ± 0.116
**DELTA G solv**	18.59 ± 0.09	10.82 ± 0.11	5.56 ± 0.07	9.84 ± 0.08
**DELTA TOTAL**	**−32.86 ± 0.10**	**−23.41 ± 0.15**	**−34.29 ± 0.07**	**−27.67 ± 0.12**

## Supplementary Materials

The following supporting information can be downloaded at: https://www.mdpi.com/article/10.3390/molecules27217317/s1, Figure S1: A superimposed structure of the apo-RBD of Deltacron at different time intervals.

## Sample Availability

Samples of the compounds are available from the authors.
