# Supplementary material for: Ultra-Large-Scale Screening of Natural Compounds and Free Energy Calculations Revealed Potential Inhibitors for the Receptor-Binding Domain (RBD) of SARS-CoV-2
Source: Molecules. 2022 Oct 28;27(21):7317. doi: 10.3390/molecules27217317 (PMC9656483; doi:10.3390/molecules27217317)
Supplement: Supplementary file 1 [file molecules-27-07317-s001.zip › molecules-1936392-supplementary.pdf]

Supplementary Materials
Ultra-large scale screening of natural compounds and free energy calculations revealed potential inhibitors for the receptor-binding domain (RBD) of SARS-CoV-2

Lisha Guo [1,†], Faryar Zafar [2], Nawal Moeen [3], Fahad M. Alshabrmi [4], Junqi Lin [5,†], Syed Shujait Ali [6], Muhammad Munir [7], Abbas Khan [8,9,*] and Dongqing Wei [7,8,9,10,11,*]

1 Zhongjing Chinese Medicine College, Nanyang Institute of Technology, 80 Changjiang Road, Nanyang 473004, China
2 Nishtar Medical University, Multan, Punjab 59341, Pakistan
3 Nawaz Sharif Medical College, Gujrat, Punjab 59341, Pakistan
4 Department of Medical Laboratories, College of Applied Medical Sciences, Qassim University, Buraydah 51452, Saudi Arabia
5 School of Biology and Biological Engineering, South China University of Technology, Guangzhou 510006, China
6 Center for Biotechnology and Microbiology, University of Swat, Swat, Khyber Pakhtunkhwa, Pakistan
7 Division of Biomedical and Life Sciences, Lancaster University, UK
8 Department of Bioinformatics and Biological Statistics, School of Life Sciences and Biotechnology, Shanghai Jiao Tong University, Shanghai 200240, China
9 Zhongjing Research and Industrialization Institute of Chinese Medicine, Zhongguancun Scientific Park, Meixi, Nayang 473006, China
10 Peng Cheng Laboratory, Vanke Cloud City Phase I Building 8, Xili Street, Nashan District, Shenzhen 518055, China
11 State Key Laboratory of Microbial Metabolism, Shanghai-Islamabad-Belgrade Joint Innovation Center on Antibacterial Resistances, Joint Laboratory of International Laboratory of Metabolic and Developmental Sciences, Ministry of Education and School of Life Sciences and Biotechnology, Shanghai Jiao Tong University, Shanghai 200030, China
* Correspondence: abbaskhan@sjtu.edu.cn (A.K.); dqwei@sjtu.edu.cn (D.W.)
† These authors contributed equally to this work.

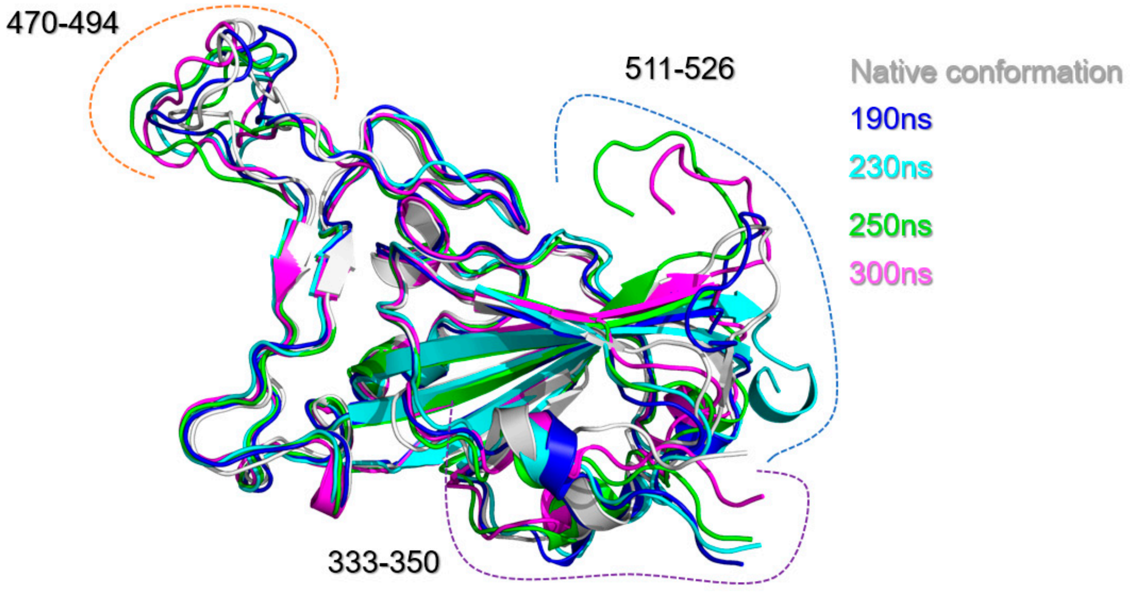

Figure S1: A superimposed structure of the apo RBD from Deltacron at different time intervals.
